# Breast cancer screening: updated recommendations of the Brazilian College of Radiology and Diagnostic Imaging, Brazilian Breast Disease Society, and Brazilian Federation of Gynecological and Obstetrical Associations

**DOI:** 10.1590/0100-3984.2017-0069

**Published:** 2017

**Authors:** Linei Augusta Brolini Dellê Urban, Luciano Fernandes Chala, Selma di Pace Bauab, Marcela Brisighelli Schaefer, Radiá Pereira dos Santos, Norma Medicis de Albuquerque Maranhão, Ana Lucia Kefalas, José Michel Kalaf, Carlos Alberto Pecci Ferreira, Ellyete de Oliveira Canella, João Emílio Peixoto, Heverton Leal Ernesto de Amorim, Helio Sebastião Amâncio de Camargo Junior

**Affiliations:** 1 Coordinator of the National Mammography Commission, Colégio Brasileiro de Radiologia e Diagnóstico por Imagem (CBR), São Paulo, SP, Brazil.; 2 Member of the National Mammography Commission, Representative of the Colégio Brasileiro de Radiologia e Diagnóstico por Imagem (CBR), São Paulo, SP, Brazil.; 3 Member of the National Mammography Commission, Representative of the Sociedade Brasileira de Mastologia (SBM), São Paulo, SP, Brazil.; 4 Member of the National Mammography Commission, Representative of the Federação Brasileira das Associações de Ginecologia e Obstetrícia (Febrasgo), Rio de Janeiro, RJ, Brazil.

**Keywords:** Breast cancer screening, Mammography, Ultrasound, Magnetic resonance imaging

## Abstract

**Objective:**

To present the current recommendations for breast cancer screening in Brazil, as devised by the Brazilian College of Radiology and Diagnostic Imaging, the Brazilian Breast Disease Society, and the Brazilian Federation of Gynecological and Obstetrical Associations.

**Materials and methods:**

We analyzed scientific studies available in the Medline and Lilacs databases. In the absence of evidence, the recommendations reflected the consensus of a panel of experts.

**Recommendations:**

Annual mammography screening is recommended for women 40-74 years of age. Among women ≥ 75 years of age, annual mammography screening should be reserved for those with an expected survival > 7 years. Complementary ultrasound should be considered for women with dense breasts. Complementary magnetic resonance imaging is recommended for women at high risk. When available, an advanced form of mammography known as tomosynthesis can be considered as a means of screening for breast cancer.

## Abstract (pt)

**Objetivo:**

Apresentar as recomendações do Colégio Brasileiro de Radiologia e Diagnóstico por Imagem, da Sociedade Brasileira de Mastologia e da Federação Brasileira das Associações de Ginecologia e Obstetrícia para o rastreamento por imagem do câncer de mama no Brasil.

**Materiais e métodos:**

Foram analisados os estudos científicos disponíveis nas bases científicas Medline e Lilacs. Na ausência de dados probatórios, as recomendações refletiram o consenso da comissão de especialistas.

**Recomendações:**

O rastreamento mamográfico anual é recomendado para as mulheres entre 40 e 74 anos. Acima de 75 anos deve ser reservado para as mulheres que tenham expectativa de vida maior que 7 anos. O rastreamento complementar com ultrassonografia deve ser considerado para as mulheres com mamas densas. O rastreamento complementar com ressonância magnética é recomendado para as mulheres com alto risco. A tomossíntese é uma forma de mamografia que pode ser considerada para o rastreamento do câncer de mama, quando disponível.

## INTRODUCTION

In a number of countries, organized screening programs have led to a reduction in breast cancer mortality^(1,2)^. In Brazil, despite all efforts, there has been an increase in the incidence of and mortality associated with breast cancer^(3-5)^. One peculiarity of breast cancer in Brazil and in other developing countries is that its incidence in women between 40 and 50 years of age is proportionately higher than that reported for developed countries^(6-8)^.

Programs that aim to standardize breast cancer screening guidelines, as well as to educate the population regarding the importance of such screening, should be promoted. In 2012, the Colégio Brasileiro de Radiologia e Diagnóstico por Imagem (CBR), the Sociedade Brasileira de Mastologia (SBM), and the Federação Brasileira das Associações de Ginecologia e Obstetrícia (Febrasgo), via the Brazilian National Mammography Commission, published their joint recommendations for breast cancer screening in Brazil^(9)^.

The purpose of this article is to present an update of those recommendations, based on the most recent and relevant scientific data on the subject.

## METHODOLOGY

To answer the clinical question "What impact do mammography, ultrasonography, magnetic resonance, and tomosynthesis have on breast cancer screening according to age bracket and personal and family risk?", we analyzed studies available via the Medline and Lilacs databases. The evaluation was based on the levels of scientific evidence established by the Oxford Centre for Evidence-based Medicine^(10)^ and on the criteria employed in the Grading of Recommendations Assessment, Development, and Evaluation approach^(11)^. In the absence of evidence, the recommendations reflect the consensus of a expert committee composed of CBR, SBM, and Febrasgo members.

The recommendations were classified into four categories, according to the degree of scientific evidence and the consensus of the specialists, as follows:

**Category A** - Recommendation based on strong scientific evidence, with a consistent consensus among the CBR, SBM, and Febrasgo that this recommendation should be strongly supported.

**Category B** - Recommendation based on reasonable scientific evidence, with a consistent consensus among the CBR, SBM, and Febrasgo that this recommendation should be strongly supported.

**Category C** - Recommendation based on minimal scientific evidence, although with a consensus among the CBR, SBM, and Febrasgo that this recommendation should be strongly supported.

**Category D** - Recommendation based on a consensus among the CBR, SBM, and Febrasgo that this recommendation should be supported.

These recommendations will be reviewed every three years.

## RECOMMENDATIONS FOR BREAST CANCER SCREENING

### SCREENING FOR BREAST CANCER IN WOMEN WITHIN THE POPULATION AT AVERAGE RISK

#### Mammography

- For women between 40 and 74 years of age, annual screening with mammography, preferably digital mammography, is recommended (*category A recommendation*).
- Among women 75 years of age or older, annual screening with mammography, preferably digital mammography, is recommended for those with an expected survival > 7 years, depending on comorbidities (*category D recommendation*).

#### Ultrasound

- There are no data to support the use of ultrasound breast cancer screening for all women within the population at average risk.
- Ultrasound should be considered as an adjunct to mammography in women with dense breasts (*category B recommendation*).

#### Magnetic resonance imaging

- There are no data to support breast cancer screening with magnetic resonance imaging for women within the population at average risk.

#### Tomosynthesis

- It is recommended that tomosynthesis be considered in association with digital mammography (COMBO or synthesized) in the screening, when available (*category B recommendation*).

### SCREENING FOR BREAST CANCER IN WOMEN AT HIGH RISK

#### Mammography

- Women with BRCA1 or BRCA2 gene mutations should undergo **annual breast cancer screening with mammography from age 30 onward**, as should women who have first-degree relatives with a proven mutation (*category B recommendation*).
- Women with a ≥ 20% lifetime risk, as calculated with one of the mathematical models based on family history, should undergo **annual breast cancer screening with mammography starting 10 years before the age at diagnosis of the youngest relative, although not before the age of 30** (*category B recommendation*).
- Women with a history of irradiation of the chest between 10 and 30 years of age should undergo **annual breast cancer screening with mammography from the 8th year after radiotherapy onward, although not beginning before the age of 30** (*category C recommendation*).
- Women diagnosed with genetic syndromes that increase the risk of breast cancer (such as Li-Fraumeni syndrome and Cowden syndrome) should undergo **annual breast cancer screening with mammography from diagnosis onward, although not beginning before the age of 30**, as should women who have first-degree relatives that have been affected (*category D recommendation*).
- Women with a history of atypical lobular hyperplasia, lobular carcinoma *in situ*, atypical ductal hyperplasia, ductal carcinoma *in situ*, or invasive breast carcinoma should undergo **annual breast cancer screening with mammography from diagnosis onward** (*category C recommendation*).

#### Magnetic resonance imaging

- Women with BRCA1 or BRCA2 gene mutations should undergo **annual breast cancer screening with magnetic resonance imaging from the age of 25 onward**, as should women who have first-degree relatives with a proven mutation (*category A recommendation*).
- Women with a ≥ 20% lifetime risk, as calculated with one of the mathematical models based on family history, should undergo **annual breast cancer screening with magnetic resonance imaging starting 10 years before the age at diagnosis of the youngest relative, although not before the age of 25** (*category A recommendation*).
- Women with a history of irradiation of the chest between 10 and 30 years of age should undergo **annual breast cancer screening with magnetic resonance imaging from the 8th year after radiotherapy onward, although not beginning before the age of 25** (*category C recommendation*).
- Women diagnosed with genetic syndromes that increase the risk of breast cancer (such as Li-Fraumeni syndrome and Cowden syndrome) should undergo **annual breast cancer screening with magnetic resonance imaging from diagnosis onward, although not beginning before the age of 25**, as should women who have first-degree relatives that have been affected (*category D recommendation*).
- Women with a history of atypical lobular hyperplasia, lobular carcinoma *in situ*, atypical ductal hyperplasia, ductal carcinoma *in situ*, or invasive breast carcinoma should undergo **annual breast cancer screening with magnetic resonance imaging from diagnosis onward** (*category C recommendation*).

#### Ultrasound

- Ultrasound should be used as a substitute for magnetic resonance imaging in women who, for any reason, cannot undergo the latter (*category B recommendation*).

#### Tomosynthesis

- It is recommended that tomosynthesis be considered in association with digital mammography (COMBO or synthesized) in the screening, when available (*category B recommendation*).

#### Justifications

The main benefit of screening is the reduction in breast cancer mortality in women over 40 years of age. To evaluate the effect of mammography screening on mortality, 11 prospective, controlled, randomized studies have been conducted^(1,2)^. Two of those studies, both conducted in Canada-Canadian National Breast Screening Study (CNBSS) 1 and CNBSS 2-had a strong selection bias^(12)^, because their study groups included a disproportionate number of patients with palpable nodules. However, the remaining studies all showed that the relative risk of death from breast cancer was lower among women who underwent mammography screening than among those who did not^(1,2)^. The study that showed the largest reduction in mortality associated with mammography screening was Swedish Two-County Trial, which reported a 31% reduction in the mammography screening group after 29 years of follow-up^(13)^. Various meta-analyses have been based on the data collected in these studies. In a meta-analysis conducted by the Independent UK Panel, the reduction in breast cancer mortality was estimated at 20%^(14)^, comparable to the 19% reported in another metaanalysis, conducted at one the Cochrane centers^(15)^.

The magnitude of the reduction in breast cancer mortality reported in the aforementioned 11 studies was questioned in a letter authored by Jorgensen et al.^(16)^. The authors placed a great deal of weight on the CNBSS studies, without considering the defects of those studies. They also argued that, because most studies of the effects of screening on breast cancer mortality were conducted in the 1960s, 1970s, and 1980s (i.e., prior to the recent therapeutic advances), the results do not reflect the current reality. They speculated that some women who were not screened and died from breast cancer would have survived if they had been treated under the current protocols. They also speculated that therapeutic advances have made early detection of breast cancer via mammography screening less relevant^(16)^. However, there is little scientific evidence to support those speculations. It is noteworthy that estimates from studies conducted in the 1970s, 1980s, and 1990s also failed to reflect the technological advances in mammography and the potential detection of more curable cancers than in the past^(17,18)^.

### SCREENING FOR BREAST CANCER BETWEEN 40 AND 49 YEARS OF AGE

Some studies have evaluated the specific impact of mammography screening for breast cancer in individuals between 40 and 49 years of age. The UK Age Trial, a prospective, controlled, randomized study, showed a 25% reduction in the relative risk of death in the first 10 years of breast cancer screening in women 39-49 years of age^(19)^. Hellquist et al.^(20)^ observed that, after 16 years of follow-up, there was a 29% reduction in mortality associated with breast cancer screening in women 40-49 years of age, whereas that reduction was 18% reduction in the subgroup of women 40-44 years of age and 32% in the subgroup of women 45-49 years of age. In an observational study conducted in Sweden, Jonsson et al.^(21)^. reported that the rate of reduction in mortality associated with breast cancer screening was 38% in women 40-49 years of age. In addition, as previously mentioned, the proportion of breast cancer patients in this age group is proportionally larger in developing countries, including Brazil, than in developed countries^(3,5)^. **Therefore, the CBR, SBM, and Febrasgo recommend that this group of women be included in breast cancer screening protocols in Brazil.**

### SCREENING FOR BREAST CANCER AT > 74 YEARS OF AGE

Prospective, controlled, randomized studies have not included women > 74 years of age, and there are therefore no direct data on screening in this age group. However, the life expectancy of women has increased, with a consequent increase in the incidence of breast cancer among women > 75 years of age. Currently, approximately 26% of breast cancer deaths occur in women diagnosed at > 74 years of age. Another factor that supports the use of mammography screening in this age group is the high sensitivity and specificity of the method^(22,23)^. Considering all of these factors, many medical organizations recommend that the decision be made on a case-by-case basis, after consulting with the patient. **Therefore, the CBR, SBM, and Febrasgo recommend that women in this age group undergo breast cancer screening if their expected survival is > 7 years.**

#### SCREENING FOR BREAST CANCER IN THE POPULATION AT HIGH RISK

When a woman is classified as being at high risk, the breast cancer screening protocol is ramped up, including two differences in relation to that applied in the general population. The first is earlier screening, because breast tumors tend to develop sooner among such women. The second is the incorporation of a complementary method (magnetic resonance imaging or ultrasound), given the limitations of mammography, which are greater in younger women.

##### Screening for breast cancer in women at high genetic risk

In women with BRCA1 or BRCA2 gene mutations, the use of supplementary screening with ultrasound or magnetic resonance imaging has been associated with the detection of a significant number of additional tumors, magnetic resonance imaging proving superior to ultrasound^(24-26)^. A systematic review published in 2007 showed that the sensitivity of mammography and ultrasound was 36% and 40%, respectively, when the methods were used separately and 55% when they were used in combination. In contrast, magnetic resonance imaging showed a sensitivity of 81% when used in isolation and 93% when combined with mammography. Therefore, the use of ultrasound as an ancillary method was found to increase the number of tumors detected, although nearly 50% of tumors still went unidentified^(27)^. Other, more recent, studies have confirmed those findings. In 2015, Riedl et al.^(28)^ reported that mammography and ultrasound both had an overall sensitivity of 38% when used separately, compared with 50% when used in combination. The authors found that magnetic resonance imaging had a sensitivity of 90% when used in isolation and 93% when combined with mammography, although there was no such increase when magnetic resonance imaging was combined with ultrasound^(28)^. However, these favorable results can be achieved only if the magnetic resonance imaging scans are of high quality, if those same scans are interpreted by physicians who are qualified to read them or at a center specializing in magnetic resonance imaging, and if it is possible to continue the investigation through biopsy of the lesions detected^(29,30)^. **Therefore, magnetic resonance imaging is the ancillary screening method of choice in women at high genetic risk, in whom ultrasound should be used only if magnetic resonance imaging, for whatever reason, cannot be performed.**

##### Other genetic syndromes

In addition to BRCA1 or BRCA2 gene mutations, there are other genetic syndromes that increase the risk for breast cancer. Such syndromes are rare, and there have been no specific studies of their relationship to screening for breast cancer. Currently, specialists recommend breast cancer screening for women with Cowden, Bannayan-Riley-Ruvalcaba, or Li-Fraumeni syndrome, as well as for untested women who have a first-degree relative with any of those syndromes^(24)^. **It is suggested that such women undergo screening in a manner similar to that recommended for women with BRCA1 or BRCA2 gene mutations.**

##### Irradiation of the chest

Women subjected to irradiation of the chest show a higher lifetime risk of developing breast cancer, comparable to the risk reported for women with BRCA gene mutations. However, the risk is variable among such women. The lifetime risk of developing breast cancer shows positive linear correlations with the radiation dose, volume of the field irradiated, and patient age at the start of treatment. Among women subjected to irradiation of the chest, mammography and magnetic resonance imaging complement each other in breast cancer screening^(31)^. Ng et al.^(32)^ reported that, among such women, the sensitivity of mammography and magnetic resonance imaging, when used separately, is 68% and 67%, respectively. However, when the two methods are used in combination, the sensitivity increases to 94%^(32)^. **Therefore, it is recommended that all patients exposed to irradiation of the chest before 30 years of age undergo screening in a manner similar to that recommended for women with BRCA1 or BRCA2 gene mutations.**

##### Atypical ductal hyperplasia and lobular neoplasia

Atypical ductal hyperplasia and lobular neoplasms (atypical lobular hyperplasia and lobular carcinoma *in situ*) are not only precursor lesions but also risk factors for breast cancer, their diagnosis increasing the relative risk of developing cancer by 4 to 10 times^(33,34)^. There is a consensus that breast cancer screening with mammography should be started soon after the diagnosis of such lesions. The great debate is regarding the use of magnetic resonance imaging in screening for breast cancer in women with such lesions. In updating its recommendations for breast cancer screening, the American Cancer Society (ACS) stated that there is no evidence to recommend or contraindicate the use of magnetic resonance imaging and that the decision regarding its use should be made on a case-by-case basis^(35)^. However, the number of advocates of the use of magnetic resonance imaging in breast cancer screening is growing.

**Therefore, it is recommended that women with atypical ductal hyperplasia or lobular neoplasia undergo screening in a manner similar to that recommended for women with BRCA1 or BRCA2 gene mutations.**

##### Personal history of breast cancer

Women with a personal history of breast cancer are at higher risk of developing a second tumor in the treated or contralateral breast^(36)^. In a recent study, the lifetime risk for the development of a second tumor was estimated to be at least 20-25%, a threshold considered by the ACS to classify women as being at high risk and to indicate complementary screening with magnetic resonance imaging^(35)^. Another study investigated the role of magnetic resonance imaging in women undergoing conservative treatment and having tested negative on mammography and ultrasound. The detection rate was 18 neoplasms per 1,000 women, which is comparable to the detection rate observed in women with BRCA gene mutations. The reported sensitivity and specificity of magnetic resonance imaging for detecting breast neoplasms in women with a personal history of breast cancer are 92% and 82%, respectively^(37)^. Other authors have reported similar values^(38)^. **Therefore, it is recommended that women who have received conservative treatment for breast cancer undergo screening with a combination of mammography and magnetic resonance imaging.**

### CONSIDERATIONS REGARDING BREAST TOMOSYNTHESIS

Tomosynthesis represents a recent step in the evolution of digital mammography, allowing more accurate evaluation of the breast. Various studies have confirmed the efficacy of tomosynthesis in screening for breast cancer, because it increases the cancer detection rate as well as reducing the false-positive rate and the recall rate^(39-41)^. The Oslo Trial was a prospective study comparing the use of the combination of tomosynthesis and digital mammography with that of digital mammography in isolation^(40)^. The authors observed that, when the combination of tomosynthesis and digital mammography was used, the cancer detection rate was 27% higher and the false-positive rate was 15% lower, with a consequent reduction in the need for invasive procedures. The STORM Trial compared digital mammography with the tomosynthesis-digital mammography combination in a sample of 7292 women^(41)^. The authors found the inclusion of tomosynthesis resulted in a 51% increase in the breast cancer detection rate and a 17% reduction in the false-positive rate. Friedewald et al.^(42)^ retrospectively analyzed 454,850 examinations, of which 281,187 were digital mammograms and 173,663 were tomosynthesis images, obtained at a total of 13 centers in the United States. The authors found that the use of tomosynthesis resulted in a 41% increase in the rate of detection of breast neoplasms, mainly primary invasive tumors, with a 15% reduction in the false-positive rate, which has the benefit of reducing screening costs. Other authors have corroborated those findings^(43,44)^.

There are still some points of contention regarding the tomosynthesis protocol. The Food and Drug Administration recommends a combined approach to breast cancer screening-digital mammography complemented with tomosynthesis (consecutively or concurrently with the digital mammography)-in which the usual digital mammography views (mediolateral oblique and craniocaudal) are combined with tomosynthesis acquisition in those same two planes. The dose of radiation, which was the main initial concern, has been shown to be lower than the maximum dose (3.0 mGy per view). Recent studies have demonstrated the efficacy of synthesized mammography, which is a new technique for digital mammography reconstruction based on the tomosynthesis images. The use of synthesized mammography maintains the benefits of tomosynthesis while reducing the dose of radiation by nearly half^(45)^. **Therefore, on the basis of data in the literature, the CBR, SBM, and Febrasgo state that tomosynthesis, when it is accessible and available, can be considered in breast cancer screening protocols, as a complement to digital mammography or as a component of synthesized mammography. These data will be reviewed every three years.**

## CONCLUSION

The reduction in breast cancer mortality, initially recorded in the United States and Europe, is the result of decades of investment focused on early diagnosis and access to appropriate treatment. Early detection of breast cancer provides benefits to women in the form of less extensive surgical procedures, an increased potential for cure, and a reduction in the ultimate costs of treatment, as well as keeping a significant portion of the female population economically active. It is fundamental that policies aimed at increasing the rate of early detection be implemented in Brazil.
